# Vasodilator therapy for pulmonary hypertension in children: a national study of patient characteristics and current treatment strategies

**DOI:** 10.1177/20458940211057891

**Published:** 2021-12-13

**Authors:** Ida Jeremiasen, Estelle Naumburg, Christian Westöö, Constance G. Weismann, Karin Tran-Lundmark

**Affiliations:** 1 Department of Experimental Medical Science and Wallenberg Centre for Molecular Medicine, Lund University, Lund, Sweden; 2 The Paediatric Heart Center, Skane University Hospital, Lund, Sweden; 3 Institution of Clinical Sciences, Paediatrics, Umeå University, Umeå, Sweden

**Keywords:** sildenafil, congenital heart defect, bronchopulmonary dysplasia, pulmonary hypertension, children

## Abstract

Pulmonary vasodilator therapy is still often an off-label treatment for pulmonary hypertension in children. The aim of this nationwide register-based study was to assess patient characteristics and strategies for pulmonary vasodilator therapy in young Swedish children. Prescription information for all children below seven years of age at treatment initiation, between 2007 and 2017, was retrieved from the National Prescribed Drug Register, and medical information was obtained by linkage to other registers. All patients were categorized according to the WHO classification of pulmonary hypertension. In total, 233 patients had been prescribed pulmonary vasodilators. The treatment was initiated before one year of age in 61% (N = 143). Sildenafil was most common (N = 224 patients), followed by bosentan (N = 29), iloprost (N = 14), macitentan (N = 4), treprostinil (N = 2) and riociguat (N = 2). Over the study period, the prescription rate for sildenafil tripled. Monotherapy was most common, 87% (N = 203), while 13% (N = 20) had combination therapy. Bronchopulmonary dysplasia (N = 82, 35%) and/or congenital heart defects (N = 156, 67%) were the most common associated conditions. Eight percent (N = 18) of the patients had Down syndrome. Cardiac catheterization had been performed in 39% (N = 91). Overall mortality was 13% (N = 30) during the study period. This study provides an unbiased overview of national outpatient use of pulmonary vasodilator therapy in young children. Few cases of idiopathic pulmonary arterial hypertension were found, but a large proportion of pulmonary hypertension associated with congenital heart defects or bronchopulmonary dysplasia. Despite treatment, mortality was high, and additional pediatric studies are needed for a better understanding of underlying pathologies and evidence of treatment effects.

## Introduction

Pulmonary hypertension (PH) is associated with considerable morbidity and mortality. Treatment options for pre-capillary WHO group 1 PH, pulmonary arterial hypertension (PAH), have increased over the last two decades, and modern therapy has improved survival but is still far from curative. Strategies for pulmonary vasodilator therapy in children is often off-label, and especially for non-group 1 PH it is in many cases based on experience and expert opinion rather than on evidence from studies.^1^

Caution is also needed when extrapolating PH treatment recommendations from adults to the pediatric population as PH etiology as well as pharmacodynamics and pharmacokinetics differ. A pediatric classification of PH, the Panama classification, was introduced in 2011.^2^ In 2013, pediatric etiologies were incorporated into the adult World Health Organization (WHO) classification of PH to create a comprehensive common classification for both adults and children.^3^ The updated version from 2018 included minor changes for better understanding and sorting of PH.^1,4,5^

The etiologies, prevalence and outcome of PH in children have been described in national register-based studies from the Netherlands and Spain.^6,7^ Pediatric data has also been published from the American REVEAL (Registry to Evaluate Early And Long-term PAH Disease Management), as well as from the international pediatric TOPP registries (Tracking Outcomes and Practice in Pediatric Pulmonary Hypertension).^8,9^ In those previous publications, patients have been included based on PH diagnosis and treatment either within a national center for PH or at a center which is linked to a particular register.

Since 2005, Sweden has a national drug register which covers all prescriptions, the Swedish Prescribed Drug Register, held by the National Board of Health and Welfare.^10^ By use of this register, it is possible to retrieve information on pediatric prescriptions of pulmonary vasodilators and through linkage to other national health registries information on underlying PH diagnosis, co-morbidities, concomitant therapies, catheterizations and mortality for each patient is accessible.

The aim of this study was to map nationwide prescription patterns and patient characteristics among young children treated with pulmonary vasodilators in Sweden.

## Materials and methods

### Study population and data collection

This national register-based study includes data on all pediatric patients younger than seven years of age at inclusion, who were born in Sweden and who received at least one prescription of a pulmonary vasodilator, for outpatient use, between 2007 and 2017. Information on prescriptions with ATC code (sildenafil ATC G04BE03, tadalafil ATC G04BE08, bosentan ATC C02KX01, ambrisentan ATC C02KX02, iloprost ATC B01AC11, treprostinil ATC B01AC21, macitentan ATC C02KX04, riociguat ATC C02KX05, vardenafil ATC G04BE09, avanafil ATC G04BE10) was retrieved from the Swedish Prescribed Drug Register.^11^ Perinatal information (gestational age) and diagnoses of primary and secondary pulmonary hypertension and related comorbidities were retrieved for each patient from the Swedish Medical Birth Register and the Swedish National Patient Register classified according to the International Statistical Classification of Diseases (ICD-10) (I2, I31, I51, P05, P07.0-07.3, P22.1, P26-27, P29.3B, Q33, Q79.0, Q90, Q20-28, I35).^12^ Two investigators (IJ and KTL) independently stratified the included patients according to the 2018 WHO classification of pulmonary hypertension.^1^ Duration of treatment, more or less than one year, was obtained for patients who initiated sildenafil therapy, the most commonly used vasodilator, in 2007–2016. Mortality data until the end of 2018 was retrieved from the Swedish Death Register. Patient and public involvement was not applicable because of the retrospective nature of the study.

### Classification of PH

The WHO classification of pediatric PH defines five main groups: (1) pulmonary arterial hypertension (PAH) including the subgroups idiopathic PAH, PAH due to congenital heart defects (CHD) with shunt and persistent pulmonary hypertension of the newborn, (2) PH due to left heart disease, (3) PH due to lung disease and/or hypoxia including the subgroups PH due to bronchopulmonary dysplasia, diaphragmatic hernia and other developmental lung disorders, (4) PH due to pulmonary artery obstructions and (5) PH with unclear multifactorial mechanisms including the subgroup PH due to complex CHD.^13^ The 2018 classification was used for this study, and therefore complex CHD was included in group 5.^1,5^ Patients for whom ICD code was missing could not be classified into a WHO group, but were stratified to a group which was named “unknown.”

### Registers

All registries used in this study are held by the Swedish National Board of Health and Welfare.^10^ A unique 12-digit national identification number is assigned to each resident in Sweden at birth and used in all official population-based registries.^14^ This identification number makes linkage between the registers possible. Data was de-identified for the researchers.

The Swedish Prescribed Drug Register was established in 2005. Information on all prescribed pulmonary vasodilators dispensed at pharmacies for each patient with dates for prescription, expedition and dosing was retrieved. Information on concomitant treatments, including diuretics, anticoagulants, antacids, pulmonary inhalations, antiarrhythmics and thyroid hormones, was also obtained.

The Swedish Medical Birth Register includes information on maternal medical history, pregnancy, delivery and data on the newborn child. Information on more than 99% of all births in Sweden, since 1973, is included. For this study, the following information was collected for each patient: date of birth, gender, gestational age at birth and information on neonatal diseases and malformations.

The Swedish National Patient Register contains data on more than 99% of patients subjected to inpatient care, and 80% of all hospital-based outpatient specialist care in Sweden and diagnoses are registered according to ICD-10 codes. Data retrieved in this study included diagnoses related to PH as described above and information on cardiac catheterization (TFC10, TFC00).

The Swedish Death Register was established in 1961. Date of death was retrieved for the purpose of this study.

### Statistical analysis

Data is presented as number (%), and median (range) as appropriate. IBM SPSS statistics Version 26 software was used for analysis.

## Results

A total of 233 pediatric patients below seven years of age met the inclusion criteria of having been treated with any of the pulmonary vasodilator medications listed. Demographics are described in Table 1. Overall, 119 boys and 114 girls were included in the study. ICD diagnosis of PH was found for 223 of 233 children and they were stratified according to WHO classification groups 1–5. The proportion of patients categorized as group 1 was 19% (N = 44), 3% (N = 6) as group 2, 46% (N = 108) as group 3 and 28% (N = 65) as belonging to group 5. No patient matched the criteria for group 4 (pulmonary hypertension due to pulmonary artery obstructions). The remaining 10 patients without ICD-10 diagnosis (4%) were stratified to the “unknown” group. Median gestational age at birth for all patients was 37 weeks (range 22–42). Almost half, 46% (N = 107), of the patients were born preterm, i.e. at a gestational age of less than 37 weeks. Among patients in WHO group 3, 74% were born preterm at a median gestational age of 27 weeks (range 22–41). Down syndrome was identified in 8% (N = 18/233) of the total study population and most commonly in WHO group 1, where 25% (N = 11/44) of the patients had Down syndrome. Heart surgery was common in the group with Down syndrome, 61% (N = 11/18), and most (82%, N = 9/11) patients had already undergone surgery when vasodilator treatment was initiated. The pulmonary vasodilator most commonly used was sildenafil, 96% (N = 224) of the total cohort 2007–2017. Duration of treatment, more or less than one year, was obtained for the patients who initiated sildenafil during 2007–2016, in total 195 patients. Of those, 64% (N = 125/195) were treated for less than one year. Concomitant medications were common and included antacids (53%), antiarrhythmics (23%), anticoagulants (18%), diuretics (70%), inhalations (71%) and thyroid hormones (6%). Cardiac catheterization was performed in 39% (N = 91/233) of all included patients and in 72% (N = 47/65) of patients in WHO group 5, the group with the most complex CHD patients.

**Table 1.** Demographics and details on treatments per WHO group.

		WHO 1	WHO 2	WHO 3	WHO 4	WHO 5	Unknown	Total
Total^a^		44 (19%)	6 (3%)	108 (46%)	0	65 (28%)	10 (4%)	233 (100%^a^)
Gender	Boys	23 (52%)	3 (50%)	56 (52%)		30 (48%)	7 (70%)	119 (51%^a^)
	Girls	21 (48%)	3 (50%)	52 (48%)		35 (52%)	3 (30%)	114 (49%^a^)
Premature birth	<37 weeks	12 (27%)	0	80 (74%)		14 (22%)	1 (10%)	107 (46%^a^)
Gestational age at birth	Weeks	38 (24–42)	39 (39–40)	27 (22–41)		39 (25–42)	38 (36–42)	37 (22–42)
Down syndrome		11 (25%)	0	2 (2%)		5 (8%)	0	18 (8%^a^)
Duration of sildenafil therapy	<1 year	22 (58%)	5 (100%)	59 (69%)		30 (53%)	9 (90%)	125 (64%)
	1 year or more	16 (42%)	0	26 (69%)		27 (47%)	1 (10%)	70 (36%)
Other medications	Antacids	21 (49%)	1 (14%)	61 (57%)		36 (55%)	4 (40%)	123 (53%^a^)
	Antiarrhythmics	12 (27%)	3 (50%)	18 (17%)		21 (32%)	0	54 (23%^a^)
	Anticoagulants	6 (14%)	0	6 (6%)		30 (46%)	0	42 (18%^a^)
	Diuretics	33 (77%)	5 (71%)	69 (64%)		55 (85%)	1 (10%)	163 (70%^a^)
	Inhalations	26 (61%)	1 (14%)	92 (85%)		40 (62%)	6 (60%)	165 (71%^a^)
	Thyroid hormones	4 (9%)	0	5 (5%)		6 (9%)	0	15 (6%^a^)
Catheterization performed		20 (47%)	1 (14%)	23 (21%)		47 (72%)	0	91 (39%^a^)

Note: Numbers are presented as n (% of WHO group) or median(range) as appropriate.

^a^Presented as n (% of total group).

**Fig. 1.**
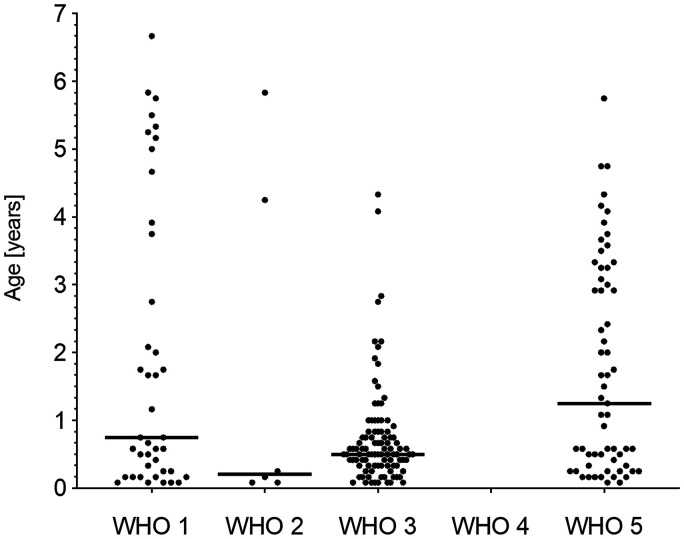
Age in years at treatment initiation per WHO group.

The median age at the first prescription of a pulmonary vasodilator was, for 61% (N = 143/233) of the patients, before the age of one year (median seven months, range 0–143 months) (Fig. 1). This was seen in all WHO groups and treatment start was particularly early in WHO group 3, with 74% (N = 80/108) before the age of one year (median six months, range 0–79 months).

**Fig. 2.**
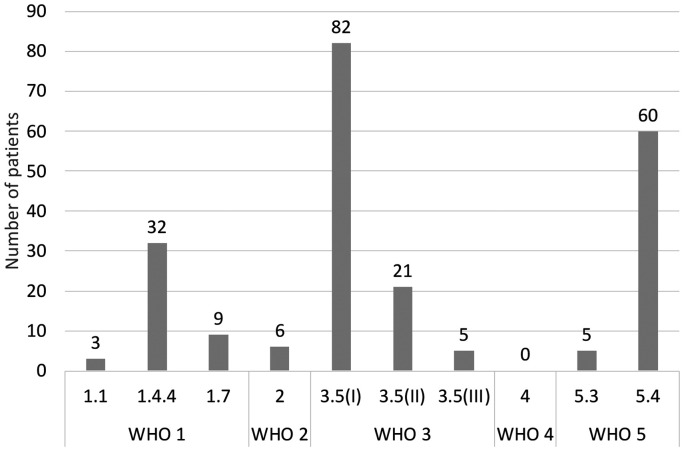
Number of patients per WHO group. Pulmonary hypertension (PH) due to: 1.1; idiopathic pulmonary arterial hypertension (PAH), 1.4.4; PAH associated with congenital heart disease (CHD), shunt, 1.7; persistent pulmonary hypertension of the newborn, 2; left heart disease, 3.5(I); bronchopulmonary dysplasia, 3.5(II); diaphragmatic hernia, 3.5(III); developmental lung disorder, 4; pulmonary artery obstructions, 5.3; other multifactorial mechanism, 5.4; complex CHD.

The classification of patients into WHO PH-subgroups is shown in Fig. 2. CHD due to shunting was the predominant subgroup within WHO group 1 with 73% (N = 32/44) while idiopathic PAH, 7% (N = 3/44), and persistent pulmonary hypertension of the newborn, 20% (N = 9/44), were uncommon causes of WHO group 1 PAH. It was not possible to investigate if any patient had a heritable form of PAH due to lack of registration of hereditary diseases in the registers. Bronchopulmonary dysplasia, 76% (N = 82/108), and diaphragmatic hernia, 19% (N = 21/108), were most common in WHO group 3 and complex CHD, 92% (N = 60/65), was the predominant cause of PH in WHO group 5. Complex CHD in WHO group 5 were distributed as follows: single ventricle 46% (N = 28/60), pulmonary atresia/significant pulmonary stenosis with or without ventricular septal defect and major aortopulmonary collaterals (MAPCAs) 17% (N = 10/60) and other complex CHD 37% (N = 22/60). In total, 67% (N = 156/233) of the patients had a CHD diagnosis, ranging from shunt to more complex CHD. Of patients with CHD, a total of 50% had undergone heart surgery, of which 65% were classified as WHO group 5, 20% to WHO group 1 and a smaller number to WHO groups 2 and 3.

Vasodilator therapies are described in Table 2, partly as total number of vasodilators per patient during the study period and partly as monotherapy or simultaneous combinations. Sildenafil was prescribed to 96% (N = 224) of all patients, as monotherapy or combination therapy. Only 4% (N = 6 bosentan and N = 3 iloprost) did not receive sildenafil at any time. The other treatments used were bosentan 12% (N = 29), iloprost 6% (N = 14), macitentan 2% (N = 4), treprostinil 1% (N = 2) and riociguat 1% (N = 2). No patient was prescribed ambrisentan, tadalafil, vardenafil or avanafil during the study period.

**Table 2.** Vasodilator therapy.

Vasodilators per patient and drugs used		Mono therapy (N)	Dual therapy (N)	Triple therapy (N)	Total (N (%))
1 vasodilator	S	194			203 (87%)
	B	6			
	I	3			
2 vasodilators	S, B	2	14		22 (9%)
	S, I		6		
3 vasodilators	S, B, I		1	2	6 (3%)
	S, B, M		2		
	S, B, T			1	
5 vasodilators	S, I, M, R, B			1	2 (1%)
	S, I, M, R, T			1	
Total (N (%))		205 (88%)	23 (10%)	5 (2%)	233 (100%)

Note: Total number of vasodilators per patient and combinations.

S: sildenafil; B: bosentan; I: iloprost; M: macitentan; T: treprostinil; R: riociguat.

Most patients were treated with only one pulmonary vasodilator (87%, N = 203/233), but 13% (N = 30/233) had a combination of multiple medications. Two patients with multiple medications, one in WHO group 1 and one in group 3, had been treated with five different pulmonary vasodilators during the study period, but not all simultaneously. Monotherapy was most common (88%, N = 205), but 10% (N = 23) of the patients were treated with dual therapy and 2% (N = 5) with triple therapy. Three of the patients with triple therapy were classified to WHO group 1, one to group 3 and one to group 5. No patient was on more than three pulmonary vasodilators simultaneously. Only sildenafil, bosentan or iloprost were used as monotherapy. All other vasodilator drugs were used in combinations. Macitentan, treprostinil and riociguat were always used as an adjunct therapy to sildenafil, bosentan or iloprost.

**Fig. 3.**
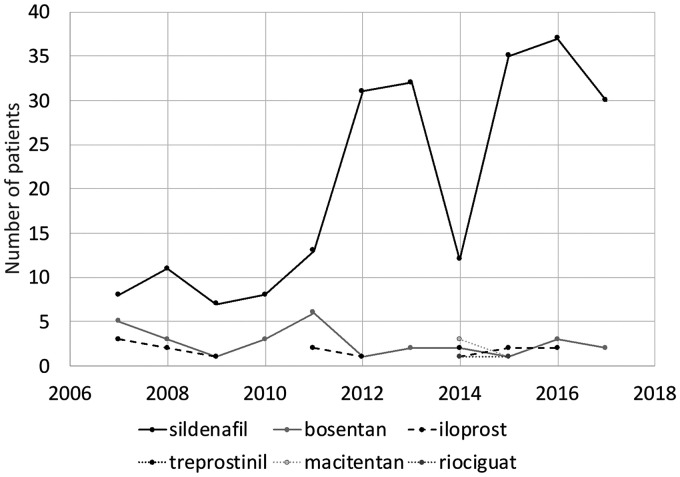
Vasodilator treatment initiation (number of patients) per year.

Prescriptions of sildenafil increased, while bosentan and iloprost were used at similar levels throughout the study period (Fig. 3). The more novel vasodilators macitentan, treprostinil and riociguat were first prescribed in 2014, and there were still only sporadic prescriptions at the end of the period.

**Fig. 4.**
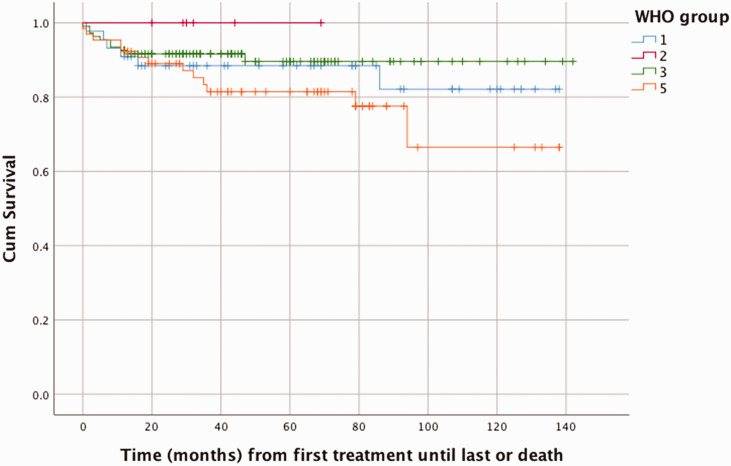
Cumulative survival per WHO group in time (months) from start of pulmonary vasodilator treatment until last treatment or death.

Overall mortality was 13% (N = 30/233) with a median age at death of two years (range 0–13) (Table 3). In WHO group 5, the group with complex CHD, the mortality was 20% (N = 13/65) and this was also the group with the highest mortality over time as seen in Fig. 4. Most patients in the deceased group had been treated with monotherapy (80%, N = 24/30), but in the group of patients who had been treated with triple therapy, the mortality rate was very high (60%, N = 3/5).

**Table 3.** Mortality data.

Total mortality	30 (13%^a^)
Gender
Boys/Girls	12 (40%)/18 (60%)
Age at death
Years	2,8/2 (0–13)
WHO group
1	6 (21%)
2	0
3	10 (34%)
5	13 (45%)
Unknown	1 (0%)
Vasodilator therapy
Single	24 (80%/12%^a^)
Dual	3 (10%/13%^a^)
Triple	3 (10%/60%^a^)

Note: Numbers are presented as n (% in mortality group/%**^a^** in total group) and for “age at death” as mean/median (range).

## Discussion

In this nationwide review of pulmonary vasodilator therapy in young pediatric patients, monotherapy with sildenafil was the most commonly used drug. A large proportion of the patients started treatment before the age of one year and belonged to the WHO PH-classification group 3 or 5, with BPD and CHD as the major associated diagnoses. Using a national prescription registry gives a new perspective, maybe a more complete picture, of the overall use of pulmonary vasodilator drug therapy in young pediatric patients, compared with studies screening for PH diagnosis or treatment used at a PH center.

PH is known to be rare in this age group. National data on PH prevalence in children of all ages has been reported by others. A Dutch study by van Loon et al. (2011) found a point prevalence of 2.2 for idiopathic PAH and 15.6 for CHD-PH per million children.^7^ A report from the Spanish national register study found a prevalence of all types of PH of 20.2 patients per million children.^6^ Ideally, the number of patients prescribed pulmonary vasodilator therapy should be in approximately the same range as the expected prevalence of all types of PH. Our data (N = 233), when adjusted for our studied age group and the population of Sweden (approximately 1 million children aged 0–7 years), suggests that the Swedish prescription rate per year is comparable to other reports. Sweden is a country with tax-financed healthcare for all citizens, and medications are free of charge for all pediatric patients. This minimizes the risk of selection bias, due to economic influence on prescription patterns and use, in our study.

### PH and associated conditions

PAH (WHO group 1 PH) was rare in our study, likely because of a low incidence in the studied age group of children below seven years of age, whereas BPD (WHO group 3) and complex CHD (WHO group 5) were more common. Neonatal care in Sweden is of high standard and is also available for the entire population because of tax-financed healthcare. One can speculate that this may contribute to a larger group of survivors discharged with BPD who may need treatment with pulmonary vasodilators.

Congenital heart defect stratified as the primary cause of PH, was common in our study and consisted of 42% of the total study population. A diagnosis of CHD, primary and secondary, was in our total cohort found in as many as 67% of the children, whereof approximately 50% had undergone heart surgery. This highlights that depending on how stratification is done numbers can also differ between studies. The Dutch study mentioned above reported 52% of PH in young patients as related to CHD and a majority were classified to WHO group 1.^7^ Pediatric patients with CHD and shunt-associated PH were classified to WHO group 1 in our study as well, but in accordance with the updated WHO classification complex CHD were categorized as part of group 5.^4^ Vasodilator therapy is regarded safe and may be effective in selected patients in this age group with complex CHD, but further studies are needed.^15,16^

Down syndrome was common in our study group (8%) and was mainly associated with CHD and shunt-related PH within WHO group 1 (25%). Cardiac surgery was performed in 61% of patients with Down syndrome. PH was diagnosed in 28% of children with Down syndrome in a study by Bush et al.^17^ They also found a majority in WHO group 1 (82%), whereof 45% was associated with CHD and 38% with persistent PH. The increased risk of PH in Down syndrome is linked to underlying CHD and respiratory challenges, and can often be treated successfully if found early.^18^ In our study, patients with Down syndrome had undergone cardiac surgery before pulmonary vasodilator treatment initiation in most cases.

### Pulmonary vasodilator treatment

The first studies on pediatric use of bosentan and sildenafil were published in the early 2000s.^19–^^21^ The most common vasodilator therapy in our study was monotherapy with sildenafil, which also gradually increased over time, with a temporary decline in 2014. In August 2012, the US Food and Drug Administration (FDA) issued recommendations, based on the STARTS-1 and at that time ongoing STARTS-2 study, which advised against the use of sildenafil for PH in children unless there was a strong indication.^22^ These recommendations were revised in 2014 based on additional information from the STARTS-2 study.^23^ One can speculate if the transient decline in Swedish prescriptions was an effect of the FDA recommendations.

In current guidelines for PH treatment in children, there is a trend towards initial upfront combination therapy. Sequential addition of complementary therapy from another class of vasodilators is also common, particularly for treatment of WHO group 1 idiopathic PAH patients.^1,13^ The patients in our study were mainly treated with monotherapy, maybe reflecting the low numbers of idiopathic PAH patients and lack of studies of combination therapy in this age group. Monotherapy with sildenafil has been reported as safe to use in pediatric PH.^24^ Also, bosentan has been reported as safe to use in pediatric patients with PAH in the repeated FUTURE study by Berger et al.^25,26^ There was not the same gradual increase of prescriptions over time for bosentan as was seen for sildenafil. Ziljstra et al. found improved survival with PAH-targeted dual and triple therapy in children with PAH (WHO group 1).^27^ Only a few selected cases within our group of young patients had dual (N = 23) or triple (N = 5) therapy. It is an attractive option to combine vasodilators, since more than one signaling pathway can be addressed and there is some support to treat also non-PAH PH.^28^ There were sporadic patients with riociguat, macitentan and treprostinil prescriptions within our study group, starting in 2014. We believe that use of macitentan and treprostinil would have been more common if teenagers had been included in this study. This study describes what was actually prescribed by Swedish doctors, during a 10-year period.

### PH assessment

Cardiac catheterization was performed in only 91 (39%) of the patients in our study, most of them with either a shunt or complex CHD (71%) or bronchopulmonary dysplasia (21%). Cardiac catheterization is a class I recommendation to assess and diagnose PH.^29^ Our results indicate that there is room for diagnostic improvement in order to accurately select candidates for pulmonary vasodilator therapy. However, catheterization can be technically difficult and hazardous in very young children with high pulmonary pressures. Median age at treatment initiation in our study was seven months, and 65% of treated patients were less than one year of age at treatment initiation. It may be justified to start vasodilator treatment in fragile patients without a prior catheterization if the diagnosis is clear by use of other diagnostic methods, such as echocardiography, and when treatment effects are monitored. Also, our study is nationwide and not only include patients from PH centers. This probably affect numbers of performed cardiac catheterizations.

### Mortality data

Mortality and morbidity in pediatric patients with PH have improved, but are still high, especially in PAH.^27^ Five-year survival of children with PAH in the REVEAL study was 74 ± 7%.^30^ Our study, with a mortality rate of 13%, is comparable with what has been reported previously. Mortality was most common in patients on triple therapy and in WHO group 5, where a majority of the patients had complex CHD. As described in Table 1, many of the children had concomitant therapies, which indicates high morbidity.

A limitation to this study is that patients aged 8–18 were not included. An important reason for not including, and sub-analyzing, older children was that the Swedish Prescribed drug Register was started in 2005 and not well established until two years later. Other limitations are the retrospective design and risk of incorrect WHO categorization, as the registry data is based on ICD-10 code diagnostics and that less than 40% of the patients were diagnosed with a catheterization, an important and major tool to diagnose PH.

In conclusion, this study provides an unbiased overview of national outpatient use of pulmonary vasodilator therapy in children younger than seven years of age at treatment initiation. A majority of children were very young (median age at first prescription was seven months) and almost half (46%) were born preterm. A catheterization rate as low as 39% is therefore not unexpected. An increasing number of prescriptions were seen over time to this group of patients with PH predominantly associated with complex CHD or bronchopulmonary dysplasia. The use of pulmonary vasodilators in this population is off-label in most cases and often based on experience and expert opinion rather that trials. It is not advisable to extrapolate results from adult studies since the patient groups and types of PH are so different.
